# Automated early detection of acute retinal necrosis from ultra-widefield color fundus photography using deep learning

**DOI:** 10.1186/s40662-024-00396-z

**Published:** 2024-08-01

**Authors:** Yuqin Wang, Zijian Yang, Xingneng Guo, Wang Jin, Dan Lin, Anying Chen, Meng Zhou

**Affiliations:** 1 National Clinical Research Center for Ocular Diseases, Eye Hospital, Wenzhou Medical University, Wenzhou, 325027 China; 2 The Affiliated Ningbo Eye Hospital of Wenzhou Medical University, Ningbo, 315042 China

**Keywords:** Acute retinal necrosis, Ultra-widefield fundus photography, Uveitis, Deep learning

## Abstract

**Background:**

Acute retinal necrosis (ARN) is a relatively rare but highly damaging and potentially sight-threatening type of uveitis caused by infection with the human herpesvirus. Without timely diagnosis and appropriate treatment, ARN can lead to severe vision loss. We aimed to develop a deep learning framework to distinguish ARN from other types of intermediate, posterior, and panuveitis using ultra-widefield color fundus photography (UWFCFP).

**Methods:**

We conducted a two-center retrospective discovery and validation study to develop and validate a deep learning model called DeepDrARN for automatic uveitis detection and differentiation of ARN from other uveitis types using 11,508 UWFCFPs from 1,112 participants. Model performance was evaluated with the area under the receiver operating characteristic curve (AUROC), the area under the precision and recall curves (AUPR), sensitivity and specificity, and compared with seven ophthalmologists.

**Results:**

DeepDrARN for uveitis screening achieved an AUROC of 0.996 (95% CI: 0.994–0.999) in the internal validation cohort and demonstrated good generalizability with an AUROC of 0.973 (95% CI: 0.956–0.990) in the external validation cohort. DeepDrARN also demonstrated excellent predictive ability in distinguishing ARN from other types of uveitis with AUROCs of 0.960 (95% CI: 0.943–0.977) and 0.971 (95% CI: 0.956–0.986) in the internal and external validation cohorts. DeepDrARN was also tested in the differentiation of ARN, non-ARN uveitis (NAU) and normal subjects, with sensitivities of 88.9% and 78.7% and specificities of 93.8% and 89.1% in the internal and external validation cohorts, respectively. The performance of DeepDrARN is comparable to that of ophthalmologists and even exceeds the average accuracy of seven ophthalmologists, showing an improvement of 6.57% in uveitis screening and 11.14% in ARN identification.

**Conclusions:**

Our study demonstrates the feasibility of deep learning algorithms in enabling early detection, reducing treatment delays, and improving outcomes for ARN patients.

**Supplementary Information:**

The online version contains supplementary material available at 10.1186/s40662-024-00396-z.

## Background

Acute retinal necrosis syndrome (ARN) is a relatively rare but highly damaging and potentially sight-threatening type of uveitis caused by human herpesvirus infection [1]. ARN initially presents as acute panuveitis, characterized by inflammation around the retinal arteries, and rapidly progresses to extensive necrotizing retinitis, often leading to rhegmatogenous retinal detachment (RRD) [2]. ARN accounts for a small proportion of uveitis cases, ranging from 0.1% to 1.3% [3–8], with an annual incidence rate of approximately 0.5 to 0.63 per million individuals [9–11]. The primary treatment approach for ARN is systemic antiviral therapy, often supplemented by intravitreal antiviral injections, effectively managing the disease [1]. However, a substantial proportion of treated eyes, ranging from 20% to 73%, still develop secondary RRD, which is the leading cause of poor visual outcomes in ARN [1, 12, 13]. Patients diagnosed with ARN who experience an average delay of 5.2 days from symptom onset to treatment are 2.3 times more likely to experience severe visual loss compared to those who receive prompt treatment within one day of symptom onset [14]. Therefore, timely and accurate diagnosis of ARN plays a critical role in ensuring effective clinical intervention and reducing the risk of permanent vision loss.

The diagnostic criteria for ARN were initially established by the Executive Committee of the American Uveitis Society in 1994, focusing on specific clinical manifestations [15]. Subsequent advances in molecular techniques have made polymerase chain reaction (PCR) testing more accessible, demonstrating high sensitivity and specificity for detecting ARN by identifying viral DNA in vitreous and aqueous specimens [10, 12, 16–18]. The Japanese ARN Study Group and the Standardization of Uveitis Nomenclature (SUN) Working Group incorporated virological testing of intraocular fluids into their classification criteria for ARN. However, these test results were not considered essential for diagnosis [19, 20]. Meanwhile, the collection of intraocular fluid is an invasive procedure with potential risks of infection. Furthermore, patients with characteristic clinical features of ARN should receive immediate antiviral treatment without waiting for the results of the PCR test. Thus, early disease detection relies on clinical expertise and subjective assessment, a significant challenge for ophthalmologists, especially in primary care settings.

Recent developments in deep learning have shown promising potential in medical image analysis [21–25]. The unique advantage of deep learning lies in its ability to discern complex and subtle features within images, enabling the identification of minute retinal changes that may escape human observation. Ultra-widefield fundus photography (UWFCFP) has been shown to be more effective than conventional fundus cameras in capturing the peripheral circumferential extension of disease [26]. To address the urgent clinical need for improved early diagnosis of ARN, we propose a deep learning model based on the Swin Transformer architecture to distinguish ARN from other types of intermediate, posterior, and panuveitis using UWFCFP. This model aims to enable computer-assisted early diagnostic tools for ARN, facilitating more accurate and timely identification of this vision-threatening disease.

## Methods

### Two-center patient cohorts

This study adhered to the tenets of the Declaration of Helsinki and was approved by the ethics committees of the Eye Hospital of Wenzhou Medical University (2023–025-K-20–01) and Ningbo Eye Hospital (2023–26(K)-C2).

A total of 1,112 subjects and 11,508 corresponding UWFCFPs [580 from normal eyes, 2,884 with ARN, and 8,044 with non-ARN uveitis (NAU)] were included in this two-center retrospective study, conducted between June 2015 to March 2023, at Eye Hospital of Wenzhou Medical University (WMUEH) and Ningbo Eye Hospital (NEH). All ophthalmic diagnoses were made by experienced uveitis and retina specialists. Normal eyes were classified based on the absence of any uveal or vitreoretinal disease, except for mild vitreous opacities or white without pressure, with no history of vitreoretinal surgery, retinal photocoagulation, and exhibiting normal fundus findings. ARN diagnosis adhered to the SUN classification criteria. Non-ARN uveitis refers to other commonly observed conditions such as intermediate, posterior, and panuveitis. Electronic medical records, multimodal imaging data, and laboratory results for each subject were independently reviewed by two ophthalmologists. Disagreements were resolved by a third uveitis specialist. A comprehensive list of disease entities and their inclusion criteria is provided in Additional file 4. Enrollment criteria for subjects required any eye to meet the outlined criteria in one of the three groups.

The photographs used in this study were obtained using a commercially available ultra-widefield (UWF) scanning laser ophthalmoscope (Daytona, Optos PLC, Dunfermline, UK) with a fixed aspect ratio of 256:325. The dataset included multiple images per patient across multiple visits, with several different images taken at different eye positions at each visit. UWFCFPs showing active inflammatory conditions were specifically selected from this dataset for the ARN and NAU cohorts. Active inflammatory conditions were identified by visual indicators such as retinal necrotic lesions, choroidal or chorioretinal lesions, and exudative retinal detachment. Exclusion criteria included the absence of inflammatory conditions and factors hindering fundus lesion observation, such as significant media opacities, intravitreal implants, retinal photocoagulation scars, and poor patient coordination during the examination. Two ophthalmologists independently reviewed each UWFCFP to ensure accurate inclusion. Disagreements were resolved through consultation with a third uveitis specialist. Specifically, 6,384 UWFCFPs were excluded based on criteria including severe media opacity (any retinal structure is completely invisible in the image, *n* = 109), presence of retinal photocoagulation scars (*n* = 1,831), quiet inflammatory period (*n* = 3,505) and interference with fundus lesion observation due to vitreous implants used in uveitis treatment (*n* = 939). Eligible photographs were then divided into four sub-cohorts for training and testing. The workflow and details of the UWFCFP collection and cohort division are illustrated in Additional file 1. Dataset volumes for each disease entity are detailed in Additional file 4.

### The architecture of deep learning algorithms

**Fig. 1:**
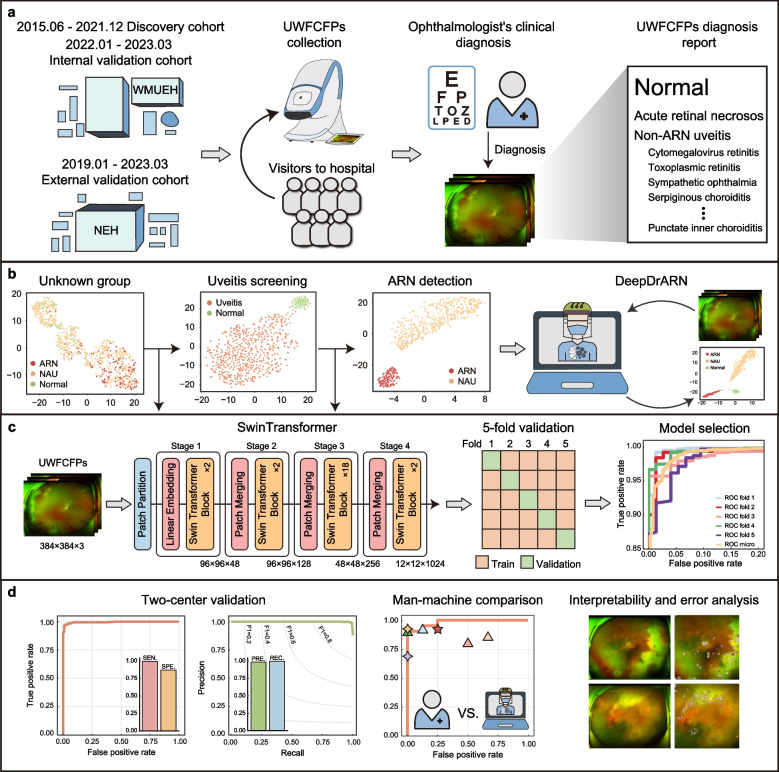
Schematic workflow of DeepDrARN. **a** Data acquisition from two ophthalmic centers in China. **b** and** c** Schematic diagram and workflow of DeepDrARN for uveitis screening and ARN identification. **d** Multi-perspective evaluation and analysis. UWFCFPs, ultra-widefield color fundus photographs; ARN, acute retinal necrosis; NAU, non-ARN uveitis; SEN, sensitivity; SPE, specificity; PRE, precision; REC, recall

The scheme of our proposed hierarchical framework, DeepDrARN, is shown in Fig. 1. DeepDrARN consists of two stages, namely uveitis screening and ARN detection. In the first stage, a deep learning model was trained to discriminate between uveitis and normal. In the second stage, the model was refined for detailed stratification, focusing on the accurate detection of ARN from NAU. We proposed a deep learning model with the Swin Transformer [27] as its backbone, which incorporates the self-attention mechanism, allowing for a comprehensive investigation of features related to ARN phenotypes. We implemented data enhancement techniques to counter color deviation and resolution disparities in UWFCFPs. UWFCFPs were resized and cropped to 384 × 384 dimensions.

We have implemented random resize cropping, augmentation, and erasing for the training set. Additionally, all RGB channels of the UWFCFPs were standardized and normalized. In the training phase, the cross-entropy loss function was used as the objective function, and the Adam optimizer was used to optimize the model. The deep learning models for uveitis screening and ARN detection were trained with a batch size of 32, a weight decay of 0.05, and learning rates of 1e − 5 and 1.25e − 4, respectively. Transfer learning was used to initialize self-attention-based deep learning architectures with parameters pre-trained on ImageNet. Five-fold cross-validation was used to ensure model robustness in the discovery cohort. Each fold was subjected to 100 training rounds, with the most accurate model saved as the best. The model with the highest accuracy among those saved in the five-fold cross-validation is selected for subsequent internal and external validation.

### Model interpretability

The integrated gradient method was used to generate pixel-level saliency maps and visual explanations for the key class-discriminative regions in the UWFCFPs as follows:

1 $$\begin{array}{c}{\text{IG}}_{i}\left(x\right)=\left(x-{x}{\prime}\right)\times {\int }_{\alpha =0}^{1}\frac{\partial F\left({x}{\prime}+\alpha \times \left(x-{x}{\prime}\right)\right)}{\partial x}d\alpha \end{array}$$

where F(x) is the deep learning model, ${\text{IG}}_{i}$ refers to the integrated gradient of pixel $i$, $x$ is the input UWFCFP and ${x}{\prime}$ is the baseline image which is a black image of the same size as the UWFCFPs.

### Statistical analysis

Statistical analyses were conducted using R software (v.4.2.2) and Python (v.3.6). Model performance was evaluated by calculating the positive predictive value (PPV), negative predictive value (NPV), accuracy, precision, recall, sensitivity, and specificity with the 'sklearn' package (v.0.24.2) at a threshold of 0.5. The area under the receiver operating characteristic curve (AUROC) and the area under the precision and recall curves (AUPR) were also calculated to assess the model’s performance. The 95% confidence intervals (CIs) for the AUROCs and AUPRs were calculated using the non-parametric bootstrap method with 2,000 resamplings with the 'pROC' package (v.1.18.0). Means and standard deviations (SDs) were used to summarize characteristics for continuous variables and percentages for categorical variables.

## Results

### Baseline characteristics of subjects and study design

A total of 5,124 UWFCFPs from 908 subjects (mean age of 42.3 ± 15.0 years; 487 men and 421 women) from two medical centers (from June 2015 to March 2023) were used to develop and validate the proposed deep learning model. Additional file 4 shows the detailed inclusion/exclusion criteria for the patients enrolled in this study. The 5,124 UWFCFPs included 580 from normal eyes, 1,000 from ARN, and 3,544 from NAU cases. Patients were divided into four sub-cohorts: (i) Discovery cohort (WMUEH-I cohort) comprising 3,533 UWFCFPs from 587 subjects at WMUEH, collected between June 2015 and December 2021 for model development; (ii) Internal validation cohort (WMUEH-II), which included 978 UWFCFPs from 235 subjects at WMUEH collected from January 2022 to March 2023; (iii) External validation cohort (NEH-I), consisting of 513 UWFCFPs from 159 subjects at NEH, collected between January 2019 and March 2023; (iv) Comparison cohort (NEH-II), consisting of the remaining 100 UWFCFPs from 66 subjects at NEH, was used for model and ophthalmologist diagnostic comparison. Demographic characteristics and clinical information of the sub-cohorts are shown in Additional file 3.

### Development and performance of the DeepDrARN

**Fig. 2:**
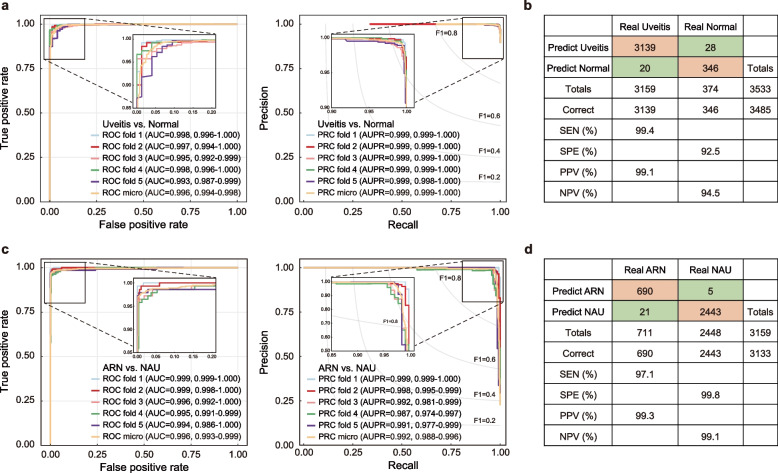
Performance of DeepDrARN in the discovery cohort. Uveitis detection with five-fold CV, ROC and PRC curves (**a**), and confusion matrix (**b**). ARN detection with five-fold CV, ROC and PRC curves (**c**), and confusion matrix (**d**). CV, cross validation; ROC, receiver operating characteristic curve; PRC, precision and recall curve; ARN, acute retinal necrosis; NAU, non-ARN uveitis; SEN, sensitivity; SPE, specificity; PPV, positive predictive value; NPV, negative predictive value

The Swin Transformer, initialized with ImageNet-trained weights, was used as the default backbone for training the DeepDrARN to effectively screen various uveitis conditions and accurately detect ARN from UWFCFPs through a five-fold cross-validation (CV) in the discovery cohort. The workflow of DeepDrARN is illustrated in Fig. 1. First, we evaluated the performance of the DeepDrARN in identifying uveitis conditions from UWFCFPs and demonstrated that it achieved AUROC, AUPRC, PPV, and NPV values of 0.996 ± 0.002, 0.999 ± 0.000, 99.1% and 94.5%, respectively (Fig. 2a and b). DeepDrARN was also evaluated for its ability to discriminate ARN from NAU. The results indicated that DeepDrARN achieved an AUROC of 0.997 ± 0.002, AUPRC of 0.993 ± 0.005, PPV of 99.3%, and NPV of 99.1% (Fig. 2c and d). Overall accuracy, precision, recall, and F1 score were analyzed for each fold (Additional file 2). The performance of DeepDrARN was consistent across data variations. These results confirmed the robustness and effectiveness in diagnosing uveitis and detecting ARN.

### Independent validation of DeepDrARN in two-center cohorts

**Fig. 3:**
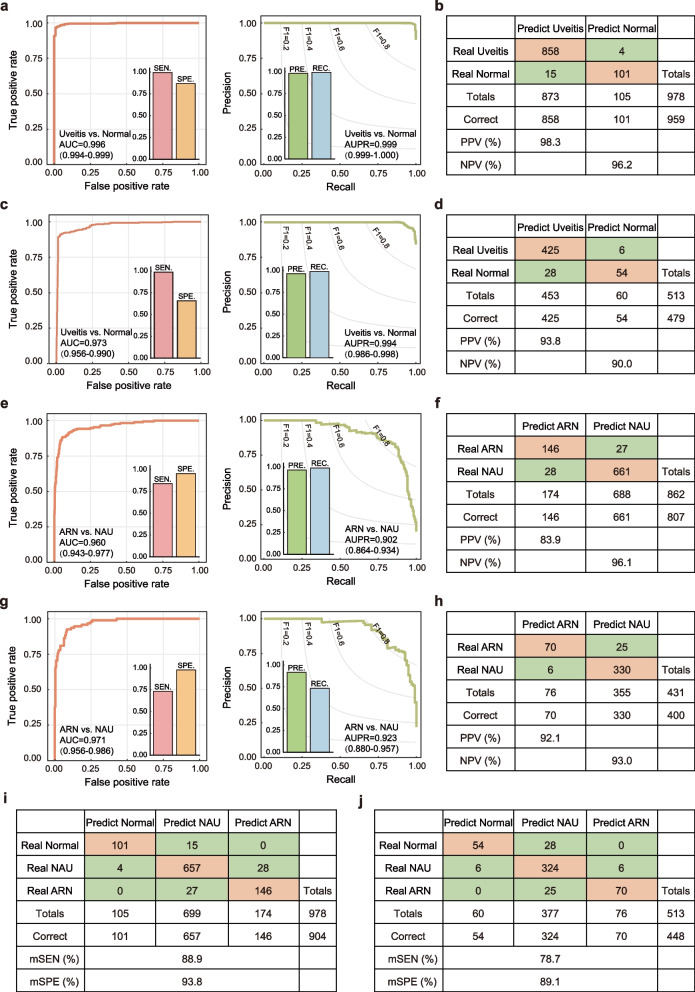
Independent evaluation of DeepDrARN. **a**–**d** ROC and PRC curves, confusion matrices for uveitis screening. **e**–**h** ROC and PRC curves, confusion matrices for ARN diagnosis. **i**, **j** Confusion matrices for differentiation of ARN, NAU, and normal subjects. ROC, receiver operating characteristic curve; PRC, precision and recall curve; ARN, acute retinal necrosis; NAU, non-ARN uveitis; AUC, area under the receiver operating characteristic curve; AUPR, area under the precision and recall curve; mSEN, mean sensitivity; mSPE, mean specificity; PPV, positive predictive value; NPV, negative predictive value; PRE, precision; REC, recall

DeepDrARN was tested in two independent cohorts from different medical centers. For uveitis screening, DeepDrARN showed similar predictive performance in two cohorts, with AUROCs of 0.996 (95% CI: 0.994–0.999) and 0.973 (95% CI: 0.956–0.990), AUPRCs of 0.999 (95% CI: 0.999–1.000) and 0.994 (95% CI: 0.986–0.998), PPVs of 98.3% and 93.8%, and NPVs of 96.2% and 90.0% for WMUEH-II and NEH-I cohorts, respectively (Fig. 3a to d). Furthermore, DeepDrARN also performed well in discriminating ARN from NAU, with AUROCs of 0.960 (95% CI: 0.943–0.997) and 0.971 (95% CI: 0.956–0.986), AUPRCs of 0.902 (95% CI: 0.864–0.934) and 0.923 (95% CI: 0.880–0.957), PPVs of 83.9% and 92.1%, and NPVs of 96.1% and 93.0% in the WMUEH-II and NEH-I cohorts, respectively (Fig. 3e to h). In addition, we tested the performance of DeepDrARN in differentiating ARN, NAU, and normal subjects from an unknown population. As shown in Fig. 3, DeepDrARN demonstrated sensitivities of 88.9% and 78.7% and specificities of 93.8% and 89.1% in the WMUEH-II and NEH-I cohorts, respectively.

### Performance comparison (DeepDrARN vs. ophthalmologists)

To further validate the diagnostic competence of DeepDrARN**,** a comparative analysis was conducted to assess its performance against seven ophthalmologists (four junior, two intermediate, and one senior). This evaluation was carried out on an independent comparison cohort (NEH-II), in which UWFCFPs had not previously been examined by either DeepDrARN or ophthalmologists.

**Fig. 4:**
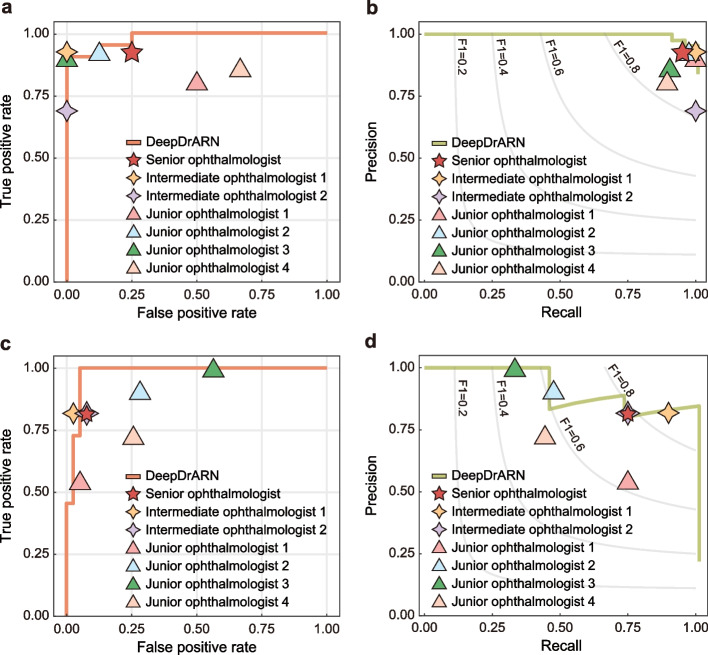
Comparison between DeepDrARN and human ophthalmologists. ROC curve (**a**) and PRC curve (**b**) for uveitis screening. ROC curve (**c**) and PRC curve (**d**) for ARN identification. ROC, receiver operating characteristic curve; RPC, precision and recall curve; ARN, acute retinal necrosis

Ophthalmologists independently and anonymously made diagnoses without patient-specific clinical information. Assessments were performed in a quiet environment without time constraints. Results of the comparative analysis are shown in Fig. 4 and Table 1. The performance of DeepDrARN was comparable to that of the ophthalmologists, and even exceeded the average accuracy of seven ophthalmologists, showing an improvement of 6.57% and 11.14% in uveitis screening and ARN identification, respectively. In contrast, considerable variation in precision and recall was observed among ophthalmologists, reflecting differences in experience and expertise, with a wide range of accuracy for both uveitis detection and ARN identification.

**Table 1:** Comparison between human ophthalmologists and DeepDrARN

Parameter	Uveitis vs. Normal				ARN vs. NAU			
	Accuracy (%)	Precision (%)	Recall (%)	F1 score	Accuracy (%)	Precision (%)	Recall (%)	F1 score
Senior ophthalmologist	90.00	95.12	92.86	93.98	90.00	94.74	92.31	93.51
Intermediate ophthalmologist 1	94.00	100.00	92.86	96.30	94.00	95.00	97.44	96.20
Intermediate ophthalmologist 2	74.00	100.00	69.05	81.69	90.00	94.74	92.31	93.51
Junior ophthalmologist 1	92.00	100.00	90.84	95.00	86.00	88.10	94.87	91.36
Junior ophthalmologist 2	92.00	97.50	92.86	95.12	76.00	96.55	71.79	82.35
Junior ophthalmologist 3	80.00	86.36	90.48	88.37	56.00	100.00	43.59	60.71
Junior ophthalmologist 4	76.00	89.47	80.95	85.00	74.00	90.63	74.36	81.69
Mean ophthalmologists	85.43	95.49	87.13	90.78	80.86	94.25	80.95	85.62
DeepDrARN	92.00	91.30	100.00	95.54	92.00	81.82	81.82	81.82

*ARN* = acute retinal necrosis; *NAU* = non-ARN uveitis

### Interpretability and misdiagnosis analysis of DeepDrARN

**Fig. 5:**
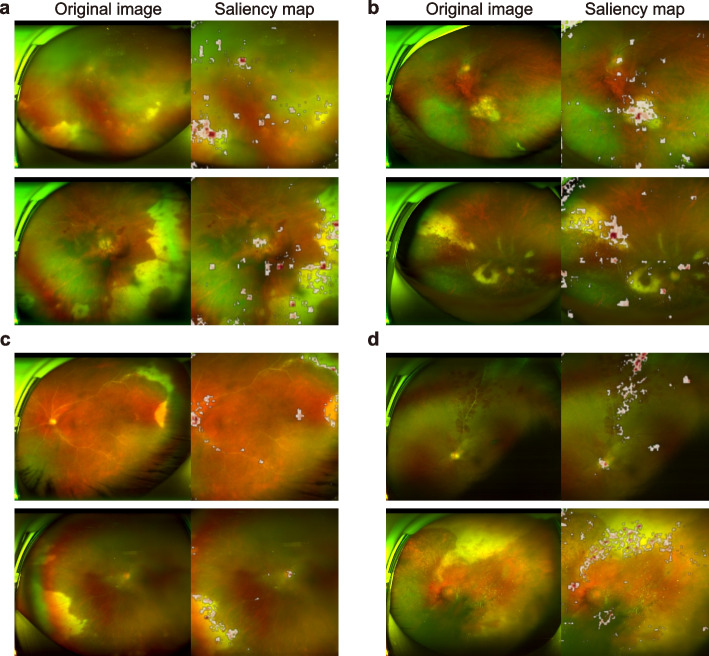
Visualization of DeepDrARN decision. Original UWFFP and saliency maps for ARN (**a**) and NAU (**b**) in uveitis screening. Original UWFFP and saliency maps for ARN (**c**) and NAU (**d**) for ARN identification. UWFFP, ultra-widefield color fundus photograph; ARN, acute retinal necrosis; NAU, non-ARN uveitis

The misdiagnosis of DeepDrARN was analyzed using integrated gradients to gain a more comprehensive understanding of DeepDrARN. Figure 5 shows representative cases and their corresponding saliency maps of DeepDrARN. The saliency maps show that DeepDrARN focuses on specific UWFCFP regions, including the optic disc, retinal blood vessels, and lesion areas (Fig. 5). Specifically, for ARN, DeepDrARN focuses primarily on critical areas such as the optic disc, necrotic lesions, vascular occlusions, and inflammatory vitreous haze (Fig. 5a and c). Notable areas of concern include retinal lesions, vasculitis or sheathing, and inflammatory vitreous haze, particularly in cases of toxoplasma retinochoroiditis (TR) (Fig. 5b top), cytomegalovirus retinitis (CMVR) (Fig. 5b bottom and 5d bottom), and idiopathic retinal vasculitis (IRV) (Fig. 5d top). These results indicate that DeepDrARN has acquired significant features that match the clinically relevant knowledge of uveitis experts, suggesting that DeepDrARN has developed the ability to prioritize key fundus areas that are critical for uveitis diagnosis. In the WMUEH-II and NEH-I cohorts, the characteristics of the misinterpreted UWFCFPs by DeepDrARN were summarized in Additional file 5. False negatives in uveitis screening were consistently observed in cases with minor or mild lesions, while false positives correlated with mild vitreous opacity (5 photographs, 33.3%) in the WMUEH-II cohort and camera lens reflections (11 photographs, 39.3%) in the NEH-I cohort. Misclassification was particularly evident during the regression phase of retinal necrosis, with 66.7% in WMUEH-II and 64.0% in NEH-I. DeepDrARN tended to misclassify specific uveitis subtypes, such as IRV and CMVR, as ARN.

## Discussion

ARN, a potentially devastating ocular disease, has a low incidence rate, leading to its underdiagnosis and misdiagnosis due to limited familiarity among many ophthalmologists in clinical practice. This study develops and validates a clinical-level deep learning model using UWFCFPs to automatically detect uveitis conditions and further differentiate ARN from other uveitis types. Early detection of ARN is crucial for preventing irreversible vision loss. Traditionally, the diagnosis of ARN relies heavily on clinical expertise, subjective assessment, and sometimes invasive procedures. These limitations highlight the urgent need for objective, non-invasive diagnostic tools, particularly in primary care. Delays caused by traditional diagnostic methods in primary care can lead to significant consequences. Growing evidence shows that integrating deep learning algorithms into clinical practice could revolutionize healthcare by improving disease diagnosis, treatment selection, and clinical laboratory testing [28–33]. Existing deep-learning retinal disease screening is primarily based on fundus images with a limited 45° to 55° field of view [34–36]. Uveitis, particularly ARN, presents unique challenges as early lesions may occur in the peripheral retina. The 200° coverage of UWFCFPs, which does not require pupil dilation, overcomes the limitations of traditional systems, making it ideal for large-scale screening. We designed a hierarchical vision transformer architecture to accurately identify disease-specific discriminative features, including subtle abnormalities in early-stage UWFCFPs.

Several recent studies have developed diagnostic models for various forms of uveitis based on clinical cases [37–40]. However, these models are unsuitable for comprehensive screening as they rely heavily on extensive clinical data for diagnosis. Conversely, existing fundus image screening models for uveitis are limited by single-center focus, small dataset size, and lack of external validation [35, 41]. This two-center retrospective study utilized the largest ARN UWFCFP dataset to date, thereby increasing the reliability and applicability of DeepDrARN. To ensure data representativeness, our study followed current international standards for uveitis diagnosis and classification, distinguishing it from previous studies that relied solely on labeling by ophthalmologists at their respective centers. Our comparative study showed that DeepDrARN matched expert performance and outperformed primary ophthalmologists in ARN detection. The reduced sensitivity and specificity of primary eye care practitioners in detecting ARN reflect their limited familiarity with this rare condition. Saliency maps allowed DeepDrARN to identify critical features, demonstrating its reliability as a diagnostic tool.

Although our study has made some efforts, certain challenges and avenues for future investigation should be acknowledged. First, further validation studies in diverse cohorts and populations are needed. Second, the retrospective nature of the study highlights the need for prospective cohorts to verify the reliability of DeepDrARN in real-world clinical settings. Furthermore, although the deep learning model has shown promise as a diagnostic aid, it is undeniable that uveitis diagnosis requires a combination of medical history, laboratory findings, and multimodal imaging rather than solely relying on a single imaging modality. Especially in cases of highly atypical or media opacity, the effectiveness of the deep learning model may be limited. Therefore, the integrated decision-making of clinical ophthalmologists and PCR testing remain indispensable and central components of the diagnostic process.

## Conclusions

This study introduces DeepDrARN, a deep learning model for automated early detection of ARN using UWFCFPs. The robust performance and non-invasive nature establish DeepDrARN as a valuable screening tool for uveitis and ARN, aiding clinical decision-making, especially for junior ophthalmologists. The potential implementation of DeepDrARN across clinical platforms shows promise in enabling early referrals, reducing treatment delays, and improving outcomes for ARN patients.

### Supplementary Information

Additional file 1. Workflow of ultra-widefield color fundus photograph (UWFCFP) collection and cohort division. ARN, acute retinal necrosis; NAU, non-ARN uveitis; WMUEH, Eye Hospital of Wenzhou Medical University; NEH, Ningbo Eye Hospital.

Additional file 2. Overall accuracy, precision, recall, and F1 score for 5-fold cross validation in uveitis screening (**a**) and ARN identification (**b**).

Additional file 3. Demographic characteristics and clinical information of the four sub-cohorts for training and testing of DeepDrARN.

Additional file 4. Inclusion criteria and data volumes of all enrolled disease entities.

Additional file 5. Characteristics of the misinterpreted ultra-widefield color fundus photographs (UWFCFPs) by DeepDrARN.

## Data Availability

The images analyzed during the current study are not publicly available due to patient privacy purposes. Data access can be obtained upon reasonable request to YW (wangyuqin@eye.ac.cn). Access to the data will be restricted to non-commercial research, which removes patient-sensitive information. The source codes are available on Github: https://github.com/ZhoulabCPH/DeepDrARN

## Abbreviations

ARN: Acute retinal necrosis
AUROC: Area under the receiver operating characteristic curve
AUPRC: Area under the precision and recall curve
CV: Cross validation
CMVR: Cytomegalovirus retinitis
IG: Integrated gradient
IRV: Idiopathic retinal vasculitis
NAU: Non-ARN uveitis
NEH: Ningbo Eye Hospital
NPV: Negative predictive value
PCR: Polymerase chain reaction
PPV: Positive predictive value
RRD: Rhegmatogenous retinal detachment
SD: Standard deviation
SUN: Standardization of uveitis nomenclature
TR: Toxoplasma retinochoroiditis
UWFCFP: Ultra-widefield color fundus photography
WMUEH: Eye Hospital of Wenzhou Medical University
