# Supplementary material for: Automated early detection of acute retinal necrosis from ultra-widefield color fundus photography using deep learning
Source: Eye Vis (Lond). 2024 Aug 1;11:27. doi: 10.1186/s40662-024-00396-z (PMC11293155; doi:10.1186/s40662-024-00396-z)
Supplement: Supplementary file 1 — Additional file 1. Workflow of ultra-widefield color fundus photograph (UWFCFP) collection and cohort division. ARN, acute retinal necrosis; NAU, non-ARN uveitis; WMUEH, Eye Hospital of Wenzhou Medical University; NEH, Ningbo Eye Hospital. [file 40662_2024_396_MOESM1_ESM.docx]

**Additional file 1.** Workflow of ultra-widefield color fundus photograph (UWFCFP) collection and cohort division. ARN, acute retinal necrosis; NAU, non-ARN uveitis; WMUEH, Eye Hospital of Wenzhou Medical University; NEH, Ningbo Eye Hospital.


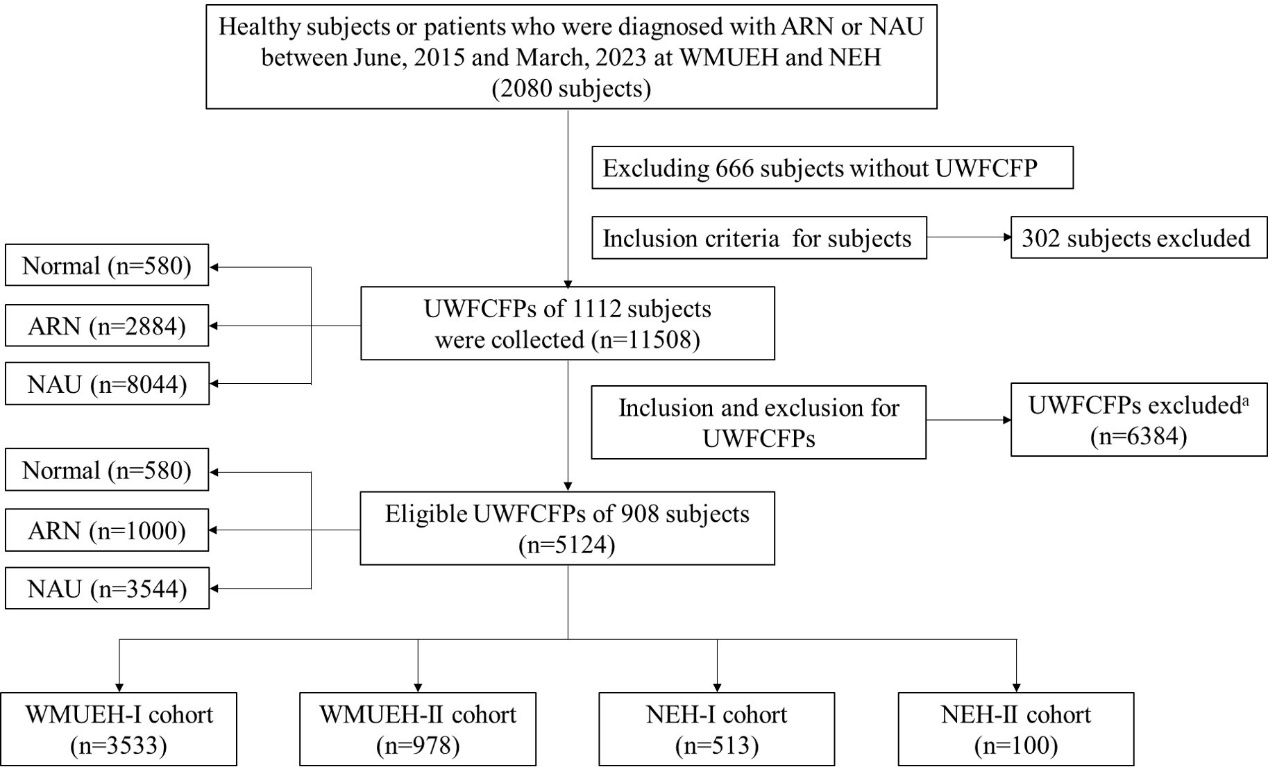


Notes: ^a^ Two hundred and four subjects with no UWFCFP qualified were excluded.
