# Supplementary material for: Automated early detection of acute retinal necrosis from ultra-widefield color fundus photography using deep learning
Source: Eye Vis (Lond). 2024 Aug 1;11:27. doi: 10.1186/s40662-024-00396-z (PMC11293155; doi:10.1186/s40662-024-00396-z)

**Additional file 2.** Overall accuracy, precision, recall, and F1 score for 5-fold cross validation in uveitis screening (**a**) and ARN identification (**b**).


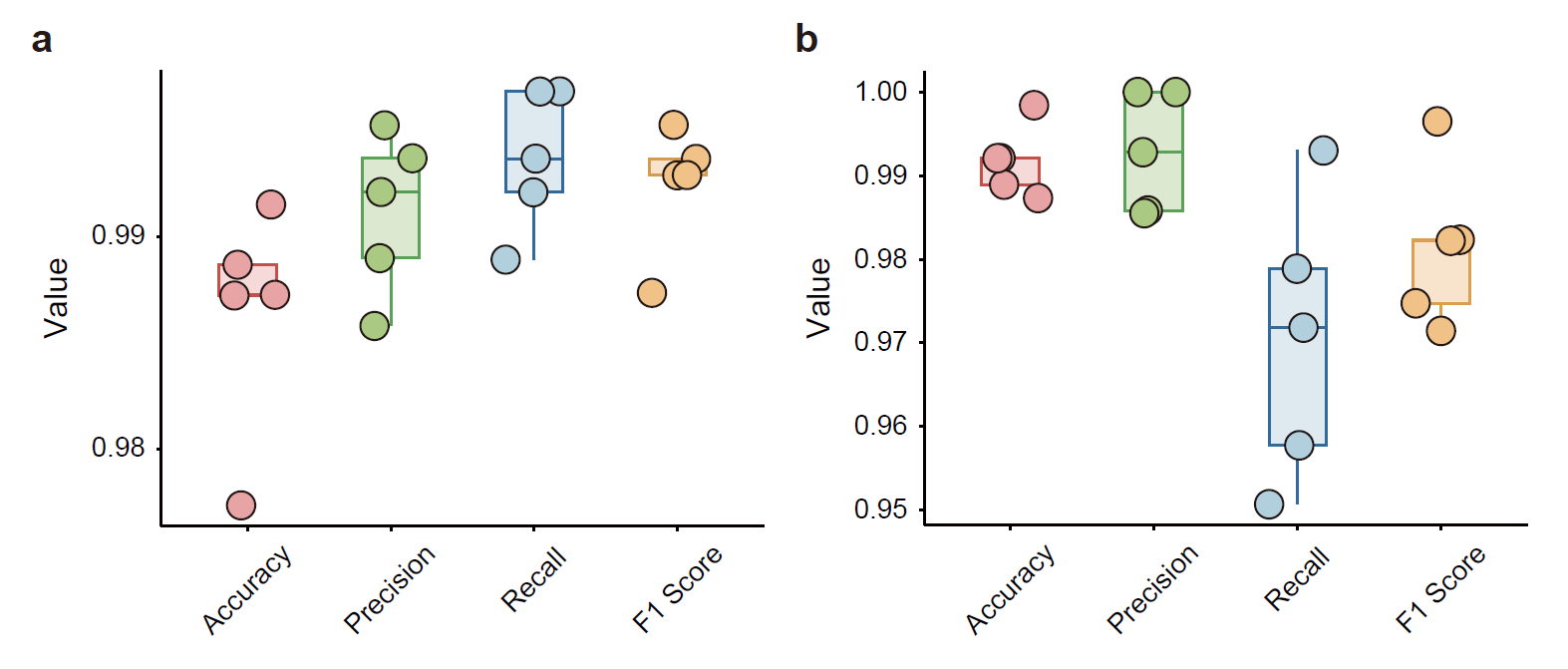

Supplement: Supplementary file 2 — Additional file 2. Overall accuracy, precision, recall, and F1 score for 5-fold cross validation in uveitis screening (a) and ARN identification (b). [file 40662_2024_396_MOESM2_ESM.docx]
