# Supplementary material for: Automated early detection of acute retinal necrosis from ultra-widefield color fundus photography using deep learning
Source: Eye Vis (Lond). 2024 Aug 1;11:27. doi: 10.1186/s40662-024-00396-z (PMC11293155; doi:10.1186/s40662-024-00396-z)
Supplement: Supplementary file 3 — Additional file 3. Demographic characteristics and clinical information of the four sub-cohorts for training and testing of DeepDrARN. [file 40662_2024_396_MOESM3_ESM.docx]

**Additional file 3. Demographic characteristics and clinical information of the** **four sub-cohorts for training and testing of DeepDrARN**

|  | WMUEH-I cohort | | | |  | WMUEH- II cohort | | | |  | NEH-I cohort | | | |  | NEH-II cohort | | | |  | Total^b^ | | | |
| --- | --- | --- | --- | --- | --- | --- | --- | --- | --- | --- | --- | --- | --- | --- | --- | --- | --- | --- | --- | --- | --- | --- | --- | --- |
|  | Normal | ARN | NAU | Total^a^ |  | Normal | ARN | NAU | Total^a^ |  | Normal | ARN | NAU | Total^a^ |  | Normal | ARN | NAU | Total^a^ |  | Normal | ARN | NAU | Total^a^ |
| Subjects | 164 | 87 | 431 | 587 |  | 81 | 21 | 152 | 235 |  | 46 | 25 | 104 | 159 |  | 8 | 16 | 43 | 66 |  | 276 | 134 | 630 | 908 |
| Age | 42.9±14.0 | 49.2±12.5 | 39.3±14.4 | 41.1±14.5 |  | 41.7±14.4 | 56.4±11.1 | 40.4±16.5 | 42.2±16.1 |  | 42.3±13.3 | 57.5±12.4 | 44.5±14.6 | 45.7±15.0 |  | 46.8±14.8 | 57.3±12.5 | 46.2±15.8 | 49.0±15.6 |  | 42.8±14.0 | 51.8±12.7 | 40.4±15.0 | 42.3±15.0 |
| Sex, female | 64 (39.0%) | 34 (39.1%) | 212 (49.2%) | 278 (47.4%) |  | 40 (49.4%) | 6 (28.6%) | 69 (45.4%) | 108 (46.0%) |  | 25 (54.3%) | 10 (40.0%) | 51 (49.0%) | 79 (49.7%) |  | 3 (37.5%) | 4 (25.0%) | 22 (51.2%) | 29 (43.9%) |  | 116 (42.0%) | 50 (37.3%) | 300 (47.6%) | 421 (46.4%) |
| Eyes | 164 | 95 | 657 | 916 |  | 81 | 23 | 217 | 321 |  | 48 | 26 | 144 | 218 |  | 8 | 16 | 53 | 77 |  | 279 | 145 | 941 | 1365 |
| Eyes, left | 83 (50.6%) | 38 (40.0%) | 322 (49.0%) | 443 (48.4%) |  | 41 (50.6%) | 15 (65.2%) | 109 (50.2%) | 165 (51.4%) |  | 21 (43.8%) | 13 (50.0%) | 74 (51.4%) | 108 (49.5%) |  | 4 (50.0%) | 7 (43.8%) | 26 (49.1%) | 37 (48.1%) |  | 136 (48.7%) | 66 (45.5%) | 467 (49.6%) | 669 (49.0%) |
| UWFCFPs | 374 | 711 | 2448 | 3533 |  | 116 | 173 | 689 | 978 |  | 82 | 95 | 336 | 513 |  | 8 | 21 | 71 | 100 |  | 580 | 1000 | 3544 | 5124 |

Note: ^a^ One hundred and thirty-two normal eyes are the fellow eyes of ARN or NAU.

^b^ There are 83 subjects simultaneously in WMUEH-I cohort and WMUEH- II cohort. There are 56 subjects simultaneously in NEH-I cohort and NEH-II cohort.

Age is shown as mean ± standard deviation.

Sex (female), left eye are shown as numbers with (%).
