# Supplementary material for: Automated early detection of acute retinal necrosis from ultra-widefield color fundus photography using deep learning
Source: Eye Vis (Lond). 2024 Aug 1;11:27. doi: 10.1186/s40662-024-00396-z (PMC11293155; doi:10.1186/s40662-024-00396-z)
Supplement: Supplementary file 4 — Additional file 4. Inclusion criteria and data volumes of all enrolled disease entities. [file 40662_2024_396_MOESM4_ESM.docx]

**Additional file 4.** Inclusion criteria and data volumes of all enrolled disease entities.

| **Entities** | **Inclusion criteria** | **No.^a^** | | |
| --- | --- | --- | --- | --- |
|  |  | **Subjects** | **Eyes** | **UWFCFPs** |
| Normal | (1) absence of uveal or vitreoretinal disease (except mild vitreous opacities or white without pressure), (2) no history of vitreoretinal surgery or retinal photocoagulation, and (3) normal funds findings | 276 | 279 | 580 |
| Acute retinal necrosis (ARN) | SUN classification criteria [1] | 134 | 145 | 1000 |
| Non-ARN uveitis (NAU) |  |  |  |  |
| Cytomegalovirus retinitis | SUN classification criteria [2] | 27 | 36 | 383 |
| Toxoplasmic retinitis | SUN classification criteria [3] | 14 | 14 | 77 |
| Vogt-Koyanagi-Harada (VKH) disease | SUN classification criteria [4] and Chinese diagnostic criteria for VKH disease [5] | 131 | 253 | 854 |
| Behçet disease uveitis | SUN classification criteria [6] | 76 | 123 | 496 |
| Sympathetic ophthalmia | SUN classification criteria [7] | 23 | 25 | 103 |
| Intermediate uveitis^b^ | SUN classification criteria [8,9] | 94 | 167 | 746 |
| Multiple evanescent white dot syndrome | SUN classification criteria [10] | 50 | 50 | 63 |
| Multifocal choroiditis with panuveitis | SUN classification criteria [11] | 5 | 6 | 32 |
| Serpiginous choroiditis | SUN classification criteria [12] | 4 | 5 | 18 |
| Punctate inner choroiditis | SUN classification criteria [13] | 36 | 42 | 57 |
| Idiopathic retinal vasculitis | (1) clinical diagnosis, (2) on evidence of systemic or ocular diseases which can cause retinal vasculitis | 137 | 179 | 510 |
| Endogenous endophthalmitis | (1) clinical diagnosis, (2) no history of any eye surgery, ocular trauma or keratitis, (3) intraocular infection of bacteria or fungus (supported by cultures or Next Generation sequencing (NGS) of intraocular fluid), or a supporting history of endogenous source [14-16] | 12 | 16 | 59 |
| Ocular Toxocariasis | (1) clinical diagnosis, (2) positive anti-Toxocara IgG in serum or aqueous humor, combined with a Goldmann–Witmer coefficient (GWC) increases [17] | 8 | 8 | 113 |
| Chorioretinitis | (1) clinical diagnosis, (2) on evidence of systemic or ocular diseases which can cause chorioretinitis | 13 | 17 | 33 |
| Total |  | 908^c^ | 1365 | 5124 |

^a^ No. refers to the number of subjects/eyes/ultra-widefield color fundus photographs (UWFCFPs) ultimately enrolled after UWFCFP screening.

^b^ Intermediate uveitis includes pars planitis and non–pars planitis type.

^c^ One hundred and thirty-two normal eyes are the fellow eyes of ARN or NAU.

**References**

[1] Standardization of Uveitis Nomenclature (SUN) Working Group.. Classification criteria for acute retinal necrosis syndrome. Am J Ophthalmol. 2021; 228:237-44.

[2] Standardization of Uveitis Nomenclature (SUN) Working Group. Classification criteria for cytomegalovirus retinitis. Am J Ophthalmol. 2021;228:245-54

[3]Standardization of Uveitis Nomenclature (SUN) Working Group. Classification criteria for toxoplasmic retinitis. Am J Ophthalmol. 2021;228:134-41.

[4] Standardization of Uveitis Nomenclature (SUN) Working Group. Classification criteria for Vogt-Koyanagi-Harada Disease. Am J Ophthalmol. 2021; 228:205-11.

[5] Yang P, Zhong Y, Du L, Chi W, Chen L, Zhang R, et al. Development and evaluation of diagnostic criteria for Vogt-Koyanagi-Harada disease. JAMA Ophthalmol. 2018;136(9):1025-31.

[6] Standardization of Uveitis Nomenclature (SUN) Working Group. Classification criteria for Behçet disease uveitis. Am J Ophthalmol. 2021;228:80-8.

[7] Standardization of Uveitis Nomenclature (SUN) Working Group. Classification criteria for sympathetic ophthalmia. Am J Ophthalmol. 2021;228:212-9.

[8] Standardization of Uveitis Nomenclature (SUN) Working Group. Classification criteria intermediate uveitis, non-pars planitis type. Am J Ophthalmol. 2021; 228:159-64.

[9] Standardization of Uveitis Nomenclature (SUN) Working Group. Classification criteria for pars planitis. Am J Ophthalmol. 2021;228:268-74.

[10] Standardization of Uveitis Nomenclature (SUN) Working Group. Classification criteria for multiple evanescent white dot syndrome. Am J Ophthalmol. 2021; 228:198-204.

[11] Standardization of Uveitis Nomenclature (SUN) Working Group. Classification criteria for multifocal choroiditis with panuveitis. Am J Ophthalmol. 2021; 228:152-8.

[12] Standardization of Uveitis Nomenclature (SUN) Working Group. Classification criteria for serpiginous choroiditis. Am J Ophthalmol. 2021;228:126-33.

[13] Standardization of Uveitis Nomenclature (SUN) Working Group. Classification criteria for punctate inner choroiditis. Am J Ophthalmol. 2021;228:126-33.

[14] Durand ML. Bacterial and fungal endophthalmitis. Clin Microbiol Rev. 2017;30(3):597-613.

[15] Bhullar GK, Dawkins RCH, Paul RA, Allen PJ. Fungal endophthalmitis: a 20-year experience at a tertiary referral centre. Clin Exp Ophthalmol. 2020;48(7): 964-72.

[16] Zhu J, Xia H, Tang R, Ng TK, Yao F, Liao X, et al. Metagenomic next-generation sequencing detects pathogens in endophthalmitis patients. Retina. 2022;42(5):992-1000.

[17] Li S, Sun L, Liu C, Wang W, Huang S, Zhang T, et al. Clinical features of ocular toxocariasis: a comparison between ultra-wide-field and conventional camera imaging. Eye (Lond). 2021;35(10): 2855-63.
