# Supplementary material for: Automated early detection of acute retinal necrosis from ultra-widefield color fundus photography using deep learning
Source: Eye Vis (Lond). 2024 Aug 1;11:27. doi: 10.1186/s40662-024-00396-z (PMC11293155; doi:10.1186/s40662-024-00396-z)
Supplement: Supplementary file 5 — Additional file 5. Characteristics of the misinterpreted ultra-widefield color fundus photographs (UWFCFPs) by DeepDrARN. [file 40662_2024_396_MOESM5_ESM.docx]

**Additional file 5.** Characteristics of the misinterpreted ultra-widefield color fundus photographs (UWFCFPs) by DeepDrARN

|  | **WMUEH-II cohort** | |  | **NEH-I cohort** | |
| --- | --- | --- | --- | --- | --- |
| **Uveitis**  **vs.**  **Normal** | **False-negative results** | **Number (%)** |  | **False-negative results** | **Number (%)** |
|  | Small or mild lesions | 4 (100.0) |  | Small or mild lesions | 6 (100.0) |
|  | **False-positive results** | **Number (%).** |  | **False-positive results** | **Number (%)** |
|  | Mild vitreous opacity | 5 (33.3) |  | Camera lens reflection | 11 (39.3) |
|  | Watered silk reflex | 4 (26.7) |  | Watered silk reflex | 9 (32.1) |
|  | Upper eye position | 4 (26.7) |  | Mild vitreous opacity | 8 (28.6) |
|  | Camera lens reflection | 2 (13.3) |  |  |  |
| **ARN**  **vs.**  **NAU** | **False-negative results** | **Number (%)** |  | **False-negative results** | **Number (%)** |
|  | Retinal necrosis in regression | 18 (66.7) |  | Retinal necrosis in regression | 16 (64.0) |
|  | Very peripheral lesions | 3 (11.1) |  | Very peripheral lesions | 9 (36.0) |
|  | Absent of necrotic foci^a^ | 3 (11.1) |  |  |  |
|  | Atypical necrotic lesion | 3 (11.1) |  |  |  |
|  | **False-positive results** | **Number (%)** |  | **False-positive results** | **Number (%)** |
|  | Misidentification of lesions | 28 (100.0) |  | Misidentification of lesions | 6 (100.0) |
|  | IRV | 19 (67.9) |  | IRV | 6 (100.0) |
|  | CMVR | 5 (17.9) |  |  |  |
|  | TR | 3 (10.7) |  |  |  |
|  | BDU | 1 (3.5) |  |  |  |

WMUEH = Eye Hospital of Wenzhou Medical University; ARN = acute retinal necrosis; NAU = non-ARN uveitis; IRV = idiopathic retinal vasculitis; CMVR = cytomegalovirus retinitis; TR = toxoplasmic retinitis; BDU = Behçet disease uveitis.

Uveitis vs. normal: positive means uveitis, negative means normal.

ARN vs. NAU: positive means ARN, negative means non-ARN uveitis (NAU).

^a^ These UWFCFPs did not capture necrotic foci but showed other abnormalities of ARN, such as vasculitis, vascular occlusion, or active inflammatory vitreous haze.
